# Out of hospital cardiac arrest outside home in Sweden, change in characteristics, outcome and availability for public access defibrillation

**DOI:** 10.1186/1757-7241-17-18

**Published:** 2009-04-17

**Authors:** Mattias Ringh, Johan Herlitz, Jacob Hollenberg, Mårten Rosenqvist, Leif Svensson

**Affiliations:** 1 Department of Cardiology, Karolinska Institutet, South Hospital, SE-118 83 Stockholm, Sweden; 2 Institute of Medicine, Dept of Molecular and Clinical Medicine, Sahlgrenska University Hospital, SE-413 45 Göteborg, Sweden; 3 Stockholm Prehospital Centre, South Hospital, SE-118 83 Stockholm, Sweden

## Abstract

**Background:**

A large proportion of patients who suffer from out of hospital cardiac arrest (OHCA) outside home are theoretically candidates for public access defibrillation (PAD). We describe the change in characteristics and outcome among these candidates in a 14 years perspective in Sweden.

**Methods:**

All patients who suffered an OHCA in whom cardiopulmonary resuscitation (CPR) was attempted between 1992 and 2005 and who were included in the Swedish Cardiac Arrest Register (SCAR). We included patients in the survey if OHCA took place outside home excluding crew witnessed cases and those taken place in a nursing home.

**Results:**

26% of all OHCAs (10133 patients out of 38710 patients) fulfilled the inclusion criteria. Within this group, the number of patients each year varied between 530 and 896 and the median age decreased from 68 years in 1992 to 64 years in 2005 (p for trend = 0.003). The proportion of patients who received bystander CPR increased from 47% in 1992 to 58% in 2005 (p for trend < 0.0001). The proportion of patients found in ventricular fibrillation (VF) declined from 56% to 50% among witnessed cases (p for trend < 0.0001) and a significant (p < 0.0001) decline was also seen among non witnessed cases.

The median time from cardiac arrest to defibrillation among witnessed cases was 12 min in 1992 and 10 min in 2005 (p for trend = 0.029). Survival to one month among all patients increased from 8.1% to 14.0% (p for trend = 0.01). Among patients found in a shockable rhythm survival increased from 15.3% in 1992 to 27.0% in 2005 (p for trend < 0.0001).

**Conclusion:**

In Sweden, there was a change in characteristics and outcome among patients who suffer OHCA outside home. Among these patients, bystander CPR increased, but the occurrence of VF decreased. One-month survival increased moderately overall and highly significantly among patients found in VF, even though the time to defibrillation changed only moderately.

## Background

Cardiovascular disease is a common cause of death in the western world and many of these deaths occur suddenly due to out-of-hospital cardiac arrest (OHCA) [1]. Survival rates in major urban areas remain poor [2], despite the introduction of the chain-of-survival concept [3] and new in-hospital treatment strategies. The use of a community-based emergency medical service (EMS) as a single rescue force may not be sufficient to improve survival, as the time from collapse to defibrillation remains long [4]. The use of automated external defibrillators (AEDs) by non-medical personnel is adding new opportunities for shortening time intervals and several EMS systems have attempted to reorganise their strategies using the "first responder concept", which involves the activation of security guards, policemen and firemen for early defibrillation [5].

The concept of Public Access Defibrillation (PAD) postulates the widespread deployment of AEDs in heavily populated areas and high OHCA incidence sites [6]. In recent years, there has been evidence of a declining incidence of OHCAs found in shockable rhythms, making fewer patients suitable for defibrillation [7,8]. This raises questions about the rationale of implementing full-scale PAD programmes. How many of all OHCA patients are really potential subjects for PAD and have their characteristics changed? In a careful analysis of the situation in Scotland in 1991 – 1998 Pell et al found 18% of all OHCA in whom CPR was attempted to be suitable for PAD.

The overall aim of this study was to describe the patients in Sweden who suffer OHCA outside home, in whom CPR was attempted during a 14 years period. The major aim was to evaluate eventual changes among these patients in characteristics and outcome with the focus on availability for PAD.

## Methods

### Swedish Cardiac Arrest Register

This survey is based on data from the Swedish Cardiac Arrest Register (SCAR). The register currently covers about 70% of all Swedish OHCA patients in whom CPR is attempted and is a quality register supported by the Swedish National Board of Health and Welfare. The figure of 70% is a rough estimation. Recent information on the representativeness of all participating centers is not available. Recent quality checks in the two largest cities (Stockholm and Göteborg) indicate that between 90–95% of patients are included in the register. A survey 9 years back indicated that the register covered between 85–90% of all cases where CPR was attempted in the participating organisations. At present we estimate that about 80% of ambulance organisations participate in the register and that about 90% of OHCA patients in each participating organisation are reported to the register. About half of all participating organisations have participated each year during the time of the survey. There is no tendency including more urban services or more rural areas during the last years. Large cities (including all major cities) and sparsely populated areas are represented in the register which has a geographical distribution covering the vast majority of Sweden. The ambulance organisations that do not report to the register are not different in terms of education or guidelines. Ambulance organisations around Sweden continuously report data and this procedure includes the completion of a standard form with a detailed description of the circumstances and interventional actions for each OHCA in which CPR was performed. The procedure is described below.

### Dispatch and ambulance organisation

There are about 100 ambulance organisations serving nine million inhabitants in Sweden. During the last few decades, the aim of the Swedish Board of Health and Welfare has been to equip every ambulance with a trained nurse and this has also gradually been implemented all over Sweden. Furthermore, an increasing number of ambulances now carry crew members with advanced training in anaesthesiology and cardiac life support.

All ambulances in Sweden are dispatched by one of 18 different dispatch centres. The dispatch centres are similar throughout the country in terms of organisation and emergency call processing. The dispatcher uses a standardised protocol with a specific questionnaire for the identified emergency. As soon as a suspected cardiac arrest is identified, the ambulance is dispatched and the emergency call proceeds. The organisation of the dispatch centres and emergency call processing has not been subject to change over the study period.

### Study design

All patients included in the SCAR suffering an OHCA in whom CPR was attempted between 1992 and 2005 were included in the study. Patients were judged to be theoretically available for PAD if the cardiac arrest took place outside the home or outside a nursing home. Bystander-witnessed and non-witnessed cases were included. Crew-witnessed cases were excluded.

For each OHCA, the ambulance crew filled in a detailed form relating to the circumstances of the arrest. The form contains information about patient characteristics such as age, gender and place of arrest (crew witnessed, at home, in a public place, in an ambulance, at work) and presumed cause of the cardiac arrest. The classification of the probable cause of the cardiac arrest was made by the ambulance crew based on information at the scene and bystander information. Their diagnosis was accepted for this study and no further checks were made. Furthermore, detailed information was included about crucial junctures at resuscitation, such as the time of collapse and the time of interventional measures such as the initiation of CPR, defibrillation, drug administration and intubation. The type of initial rhythm was registered and defined as VF (this includes pulseless ventricular tachycardia) or asystole. The form also includes EMS-related data concerning the time of ambulance dispatch and arrival at the scene. Information was entered about bystander characteristics, such as whether or not the collapse was witnessed and whether bystander CPR was performed. The outcome of resuscitation attempts was defined as dead on ambulance arrival, dead in the emergency room, admitted to hospital and survival to one month. All the data were computerised in a database in Göteborg. The content of the form, definitions and the way data were reported to the SCAR remained unchanged during the study period.

This study was approved by the local ethics committee.

### Statistical methods

Proportions are expressed as percentages and continuous variables as medians. Trend tests for associations with the time variable year of OHCA were performed using the Mann-Whitney U test for dichotomous variables and Spearman's rank correlation for continuous variables. In the evaluation of proportions Fisher's exact test was used. All p-values are two-tailed and considered significant if below 0.05.

## Results

Overall there were 38710 patients suffering OHCA in whom CPR was attempted included in the register between 1992 and 2005 of whom12% had a crew witnessed OHCA, and 62% occurred either at home or in a nursing home. The overall survival to 1 month was 5.4%.

### Patient characteristics and percentage of patients available for PAD

Twenty-six % of all OHCA patients fulfilled the inclusion criteria. The corresponding percentage values for the 3 largest cities in Sweden (Stockholm, Göteborg and Malmö) was 27% and for the remaining part of Sweden it was 26% (p = 0.03) The total number of patients included from 1992–2005 was 10133 with an annual inclusion rate that varied between 530 and 896 patients (Additional file 1, Table S1). The median age declined from 68 years to 64 years during the study period (p for trend = 0.003). The proportion of OHCAs of cardiac origin decreased from 72% in 1992 to 61% in 2005 (p for trend < 0.0001). No significant trend was found regarding sex distribution.

### Time intervals, initial rhythm, and bystanders

The median time interval from cardiac arrest to defibrillation was 12 minutes in 1992 and 10 minutes in 2005 (p for trend = 0.029); changes were minor (Additional file 1, Table S1). The ambulance response time increased (p for trend < 0.0001) but the time between cardiac arrest and start of CPR decreased (p for trend < 0.0001) (Additional file 1 Table S1).

The proportion of patients initially found in VF was analysed for three different groups of patients: all OHCA cases, bystander-witnessed cases and non-witnessed cases.

**Figure 1:**
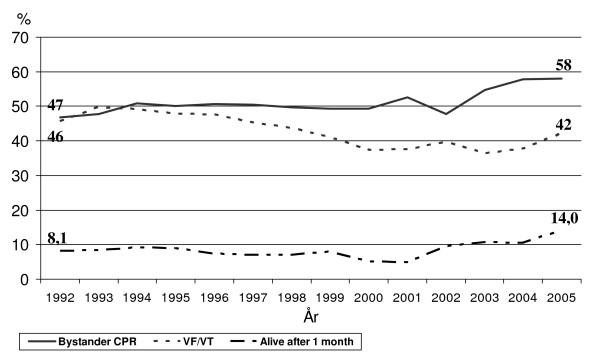
**Trend curves for changes in survival to 1 month, bystander CPR and occurrence of ventricular fibrillation**.

As shown in Additional file 1, Table S2 and Figure 1, the proportion of patients found in VF decreased significantly within all three groups.

The proportion of bystander-witnessed OHCA cases did not show any significant trend during the study period. However, a marked increase from 47% to 58% (p for trend < 0.001), in the proportion of OHCAs receiving bystander CPR was observed (Additional file 1, Table S3, Figure 1).

### Survival (Additional file 1, Table 1–3, Figure 1)

The proportion of patients admitted alive to hospital tended to increase during the study period (p for trend = 0.03). Survival to one month was analysed within five different groups of patients. Among all patients there was an increase in survival to 1 month (p for trend = 0.01). In the subgroup of patients found in VF there was a significant increase, from 15.3% in 1992 to 27.0% in 2005 (p < 0.0001 for trend), in one month survival. In Figure 1 is shown trend curves for changes in overall survival to 1 month, occurrence of ventricular fibrillation and bystander CPR.

## Discussion

### Percentage of patients available for PAD

The principal findings in this study are that about a quarter (26%) of all OHCA patients in Sweden in 1992–2005 occur outside home and are not crew witnessed and that, among these patients, there is a decreasing number of patients with VF as the first recorded rhythm despite an increasing rate of bystander CPR.

Within the study period, there were no alterations in the guidelines relating to whether or not CPR should be attempted. The conclusion is nevertheless that there was no dramatic change in the number of OHCAs that might be candidates for PAD.

In Scotland, Pell and colleagues found that 18% of all OHCAs were found to be suitable for PAD in the 1990ths. [9]. The larger percentage (26%) found in our study is explained by the wider definition, including all "theoretically" available OHCAs. Considerations based on the location of the OHCA, witnessed status or whether the OHCA was "practically" suitable for defibrillation were not taken into account in our study, whereas in the Scottish survey they excluded OHCAs on street, in train, tram etc. All cases excluded in the Scottish study might not be relevant for the situation today. For example are there plans to equip major trains in Sweden with defibrillators in the near future. Furthermore, soon will some taxidrivers in Stockholm have AED in their cars which might make PAD also in streets feasible. The proportion of patients who in reality will be available for PAD might be somewhere between 18% as in the Scottish survey and 26% as in our survey

In the study from Scotland 36% of all non crew witnessed OHCAs occurred outside home which is similar to our findings (34%). However, the proportion of patients found in a shockable rhythm appeared to be much higher in the Scottish survey as compared with our survey.

## Epidemiology

We estimate that the Swedish Out of Hospital Cardiac Arrest Register includes about 70% of all OHCA:s in whom CPR was attempted. This is due to a combination of limited number of ambulance organisations, which reported to the register and a limited number of reports from the participating organisations.

Our estimate indicate that there are about 45 OHCAs in whom CPR is attempted per 100.000 inhabitants and year. It is important to stress that these cases cover only a minority of cardiovascular deaths in Sweden (in a large proportion CPR is never started). According to statistics from the Swedish National Board of Health and Welfare there was a total number of 26132 persons who died from cardiac disease in Sweden in 2005 (289/100 000 inhabitants and year). About two thirds (n = 17709) of these deaths were due to ischemic heart disease (ICD-10, I20–I25) and one third was due to other forms of heart disease (ICD-10, I30–I52).

### Patient characteristics

We found a trend towards a decreasing median age, with a drop from 68 to 64 years during the study period. This in not line with what others have found. From a study conducted in Seattle between 1977 and 2001, Rea and colleagues reported an increase in the mean age among EMS-treated cardiac arrests from 64 to 68 years of age [10]. It is only possible to speculate that, among the victims of sudden death included in our study, there is a higher percentage of OHCAs with undiagnosed cardiac disease, physically capable and healthy enough to be out in public places. These cases perhaps conform to a higher extent with "hearts too good to die" [11]. On the other hand, Kuisma and co-workers found that OHCA of non-cardiac origin is more likely to take place among the younger members of the population and is secondary to pulmonary disease, internal bleeding, suicide, trauma and drug intoxication [12]. These findings could suggest that the drop in the mean age of victims of OHCAs in our survey could to some extent be explained by the concurrent increase in OHCAs of non-cardiac aetiology that was also observed. The data relating to the aetiology of the OHCAs in our study must be interpreted carefully, as they are based on the clinical judgement of the EMS personnel and not on autopsies or clinical investigations.

### Bystanders

We found that bystander CPR increased from 47% to 57%. These results are promising and could be the result of a greater knowledge of CPR among the general population. During the last few decades, large-scale educational efforts have been made to spread a knowledge of CPR among the Swedish population [13] and the increase in bystander CPR may be a result of these efforts. During the study period, telephone-assisted CPR was implemented in 1997. These measures may also have contributed to the overall increase in bystander CPR

### Initial rhythm

A major finding is the declining incidence of VF as the first recorded rhythm also in this cohort. The decline applies to all the patients in the study, as well as to the subgroups of bystander-witnessed and non-witnessed cases. These findings are confirmed by data reported by others and this observation has been made in both Europe and the United States [14,15]. However, it is the first time that the decline is reported among theoretical candidates for PAD during such a long follow up. A declining percentage of OHCA patients with VF as the first recorded rhythm has been observed, despite efforts to reduce call-to-shock time through PAD programmes, first responder systems and increased bystander action. Different theories have been launched to explain the declining incidence of VF. Bunch and colleagues [16] reported a decline in the incidence of VF attributed to ischemic heart disease, which suggests that successful secondary and primary prevention against ischemic heart disease are contributing to a lower incidence of OHCAs found in VF. It has been suggested that the increasing use of reperfusion therapy, smoking cessation, cardiac surgery, anti-arrhythmic and anti-thrombotic drugs, as well as implantable cardioverter defibrillators (ICD) and lipid lowering drugs, is having an impact on sudden cardiac death, since ischemic heart disease is the main cause of life-threatening arrhythmias. The widespread use of beta-blocking agents as a cornerstone in the treatment of ischemic heart disease has been proposed as an important promoter of these changes [17]. The explanations given above can also help us to understand our data. According to statistics from the Swedish National Board of Health and Welfare, the incidence, morbidity and mortality due to ischemic heart disease are decreasing sharply in Sweden and in the rest of the western world [18,19]. The call-to-shock interval has remained rather constant throughout the study period, and it can therefore hardly be used to explain these changes.

The drop in VF incidence in our material can also be partly explained by the concomitant decrease in the number of OHCAs judged to be of cardiac origin, as patients with other etiology are more likely to present as asystole or PEA. The decrease in the percentage of OHCAs judged to be of cardiac origin is probably due to the decrease in morbidity from cardiovascular disease. Data from the Swedish Death Registry state that the number of deaths from suicide, drowning, intoxication and accidents remained unchanged or decreased during the study period, suggesting that an increased number of OHCA patients suffer from "multi-system organ failure" or other chronic illnesses. [20].

### Survival

Bystander CPR and VF as the first recorded rhythm are two factors strongly associated with improved survival after OHCA [21]. One-month survival among victims of OHCAs increased particularly among patients found in ventricular fibrillation. This increase could be a result of improved post-resuscitation care following the introduction of new treatments such as mild hypothermia and early revascularisation, as well as pre-hospital improvements including an increase in bystander CPR. Improvements in pre-hospital and in-hospital factors can help to explain why overall survival to one month increased, despite the drop in the incidence of ventricular fibrillation.

### Our findings in the context of PAD and first responder programmes

The alarming evidence about a decline in the incidence of VF found among patients who suffer OHCA outside home has been confirmed by several other studies which did not particularly focus on OHCA outside home. In the light of these findings, PAD and public access programmes are likely to become less successful if this trend continues. On the other hand, shortening time intervals using first responder programmes could be the way to reverse this trend. This raises the question of the cost effectiveness of PAD programmes which has previously been debated [22]. There is good evidence to suggest that the structured, wide deployment of AEDs with trained laymen alerted by a central dispatch centre system could improve survival rates in selected populations [23]. A recent Austrian PAD study makes it clear that unstructured and "over the counter" PAD programs are probably less effective [24]. However, the question of whether it is reasonable to exclude all OHCAs that take place in non-public places can also be discussed. By doing this, total survival rates after OHCA can hardly be affected. Only survival in absolute numbers will be affected.

In spite of this, sudden cardiac death is a major health problem and one of the main causes of death. Tremendous efforts are being made in the in-hospital world to take care of patients and, as a result, most patients die outside hospital. While PAD programmes only appear to affect about 15–25% of all OHCAs, substantial progress has to be made if overall survival rates are to be affected. Perhaps we should concentrate on numbers of survivors instead of survival rates? The limitations of not reaching the majority of OHCAs that do not take place in public places are included in the PAD concept. The time intervals within the standard EMS system are still too long. New techniques could perhaps lead to the more rapid activation of first responders, making it possible to reach OHCAs earlier. Further knowledge about the changing incidence and treatment of non-shockable rhythms also needs to be generated. This will perhaps be the main challenge in the future.

## Limitations

1. There is some degree of uncertainty with regard to representativeness of the register.

2. There is missing information with regard to all variables in the register.

3. The register is not detailed enough to fully cover the "true" availability for PAD.

## Conclusion

In Sweden, 26% of all OHCAs in whom CPR was started occur outside home but are not crew witnessed and might theoretically be regarded as candidates for PAD. Among these patients, bystander CPR has increased, but the percentage found in ventricular fibrillation has decreased. Time to defibrillation has remained almost unchanged. By reducing the delay in the chain of survival, the decrease in ventricular fibrillation could be reversed. Widespread PAD programmes can play a crucial role in this health care area, although new ways to alert first responders and reach OHCA victims may be necessary if total survival rates are to be affected.

## Competing interests

The authors declare that they have no competing interests.

## Authors' contributions

MR has contributed by analysing all the data and written the manuscript. JH has contributed by preparing the design of the manuscript including tables and figures and has also critically evaluated the text and is responsible for all figures in the tables. JaH has participated in the design of the manuscript and critically evaluated the text of the manuscript. MR has participated in the design of the manuscript and critically evaluated the text of the manuscript. LS has participated in the design of the manuscript and critically evaluated the text of the manuscript. All authors read and approved the final manuscript.

## Supplementary Material

Additional file 1 **Table S1, S2 and S3**. Table S1 – Proportion of patients available for PAD and their characteristics and outcome. Table S2 – Occurrence of ventricular fibrillation, delay to defibrillation and outcome in relation to ventricular fibrillation. Table S3 – Total witnessed status, bystander CPR and outcome in relation to witnessed status. Click here for file
