# Supplementary material for: Out of hospital cardiac arrest outside home in Sweden, change in characteristics, outcome and availability for public access defibrillation
Source: Scand J Trauma Resusc Emerg Med. 2009 Apr 17;17:18. doi: 10.1186/1757-7241-17-18 (PMC2678978; doi:10.1186/1757-7241-17-18)
Supplement: Additional file 1 — Table S1, S2 and S3. Table S1 – Proportion of patients available for PAD and their characteristics and outcome. Table S2 – Occurrence of ventricular fibrillation, delay to defibrillation and outcome in relation to ventricular fibrillation. Table S3 – Total witnessed status, bystander CPR and outcome in relation to witnessed status. [file 1757-7241-17-18-S1.doc]

|  | **1992** | **1993** | **1994** | **1995** | **1996** | **1997** | **1998** | **1999** | **2000** | **2001** | **2002** | **2003** | **2004** | **2005** | **p-value*** |
| --- | --- | --- | --- | --- | --- | --- | --- | --- | --- | --- | --- | --- | --- | --- | --- |
| **Total (n)** | 2609 | 2862 | 3094 | 3184 | 3295 | 3142 | 3119 | 2216 | 2649 | 2457 | 2382 | 2417 | 2484 | 2800 |  |
| Available for PAD(n) | 737 | 787 | 850 | 896 | 859 | 824 | 814 | 530 | 689 | 635 | 590 | 608 | 614 | 701 |  |
| Available for PAD (%) | 28 | 27 | 27 | 28 | 26 | 26 | 26 | 24 | 26 | 26 | 25 | 25 | 25 | 25 |  |
| **Age** (years, median) | 68 | 69 | 69 | 68 | 68 | 69 | 69 | 69 | 68 | 67 | 67 | 68 | 65 | 64 | 0.003 |
| Non missing (n) | 668 | 714 | 771 | 822 | 781 | 767 | 751 | 506 | 599 | 572 | 526 | 533 | 548 | 606 |  |
| Missing (n) | 69 | 73 | 79 | 74 | 78 | 57 | 63 | 24 | 90 | 63 | 64 | 75 | 66 | 95 |  |
| **Gender** |  |  |  |  |  |  |  |  |  |  |  |  |  |  |  |
| Women (n) | 148 | 147 | 152 | 156 | 168 | 159 | 148 | 103 | 116 | 107 | 104 | 119 | 101 | 131 |  |
| Non missing (n) | 693 | 745 | 802 | 872 | 821 | 792 | 787 | 504 | 658 | 603 | 543 | 565 | 579 | 669 |  |
| Women (%) | 21 | 20 | 19 | 18 | 20 | 20 | 19 | 20 | 18 | 18 | 19 | 21 | 17 | 20 | > 0.2 |
| Missing (n) | 44 | 42 | 48 | 24 | 38 | 32 | 27 | 26 | 31 | 32 | 47 | 43 | 35 | 32 |  |
| **Etiology** |  |  |  |  |  |  |  |  |  |  |  |  |  |  |  |
| Cardiac (n) | 472 | 505 | 542 | 576 | 552 | 536 | 526 | 343 | 415 | 369 | 357 | 344 | 353 | 400 |  |
| Non missing (n) | 655 | 707 | 773 | 807 | 784 | 759 | 750 | 491 | 626 | 565 | 520 | 561 | 568 | 655 |  |
| Cardiac (%) | 72 | 71 | 70 | 71 | 70 | 71 | 70 | 70 | 66 | 65 | 69 | 61 | 62 | 61 | < 0.0001 |
| Missing (n) | 82 | 80 | 77 | 89 | 75 | 65 | 64 | 39 | 63 | 70 | 70 | 47 | 46 | 46 |  |

**Table 1**

**Proportion of patients available for PAD and their characteristics and outcome**

| **Delay** |  |  |  |  |  |  |  |  |  |  |  |  |  |  |  |
| --- | --- | --- | --- | --- | --- | --- | --- | --- | --- | --- | --- | --- | --- | --- | --- |
| From call to arrival of ambulance (median,min) | 5 | 5 | 5 | 6 | 5 | 6 | 6 | 6 | 5 | 6 | 6 | 6 | 7 | 7 | < 0.0001 |
| Non missing (n) | 691 | 757 | 805 | 860 | 809 | 788 | 777 | 505 | 642 | 594 | 559 | 570 | 593 | 670 |  |
| Missing (n) | 46 | 30 | 45 | 36 | 50 | 36 | 37 | 25 | 47 | 41 | 31 | 38 | 21 | 31 |  |
| From cardiac arrest to start  of CPR (median, min) | 8 | 7 | 7 | 8 | 7 | 8 | 7 | 7 | 8 | 7 | 7 | 8 | 5 | 5 | < 0.0001 |
| Non missing (n) | 346 | 381 | 453 | 446 | 426 | 405 | 414 | 279 | 338 | 285 | 280 | 299 | 311 | 342 |  |
| Missing (n)** | 391 | 406 | 397 | 450 | 433 | 419 | 400 | 251 | 351 | 350 | 310 | 309 | 303 | 359 |  |
| **Survival** |  |  |  |  |  |  |  |  |  |  |  |  |  |  |  |
| Survival to ward (n) | 146 | 169 | 171 | 198 | 183 | 140 | 152 | 87 | 127 | 116 | 116 | 140 | 145 | 187 |  |
| Non missing (n) | 729 | 780 | 845 | 892 | 855 | 823 | 812 | 530 | 689 | 632 | 556 | 585 | 589 | 695 |  |
| Survival to ward (%) | 20.0 | 21.7 | 20.2 | 22.2 | 21.4 | 17.0 | 18.7 | 16.4 | 18.4 | 18.4 | 20.9 | 23.9 | 24.6 | 26.9 | 0.03 |
| Missing (n) | 8 | 7 | 5 | 4 | 4 | 1 | 2 | 0 | 0 | 3 | 34 | 23 | 25 | 6 |  |
|  |  |  |  |  |  |  |  |  |  |  |  |  |  |  |  |
| Survival to 1 month (n) | 59 | 64 | 77 | 80 | 63 | 57 | 57 | 42 | 34 | 30 | 54 | 63 | 62 | 97 |  |
| Non missing (n) | 728 | 775 | 840 | 890 | 853 | 824 | 812 | 529 | 687 | 624 | 572 | 591 | 598 | 689 |  |
| Survival to 1 month (%) | 8.1 | 8.3 | 9.2 | 9.0 | 7.4 | 6.9 | 7.0 | 7.9 | 5.0 | 4.8 | 9.4 | 10.7 | 10.4 | 14.1 | 0.01 |
| Missing (n) | 9 | 12 | 10 | 6 | 6 | 0 | 2 | 1 | 2 | 11 | 18 | 17 | 16 | 12 |  |

* p-value denoted if < 0.2

** Number of patients with missing information including all non witnessed cases

**Table 2**

Occurrence of ventricular fibrillation, delay to defibrillation and outcome in relation to ventricular fibrillation

|  | **1992** | **1993** | **1994** | **1995** | **1996** | **1997** | **1998** | **1999** | **2000** | **2001** | **2002** | **2003** | **2004** | **2005** | **p-value*** |
| --- | --- | --- | --- | --- | --- | --- | --- | --- | --- | --- | --- | --- | --- | --- | --- |
| **VF as initial rhythm** |  |  |  |  |  |  |  |  |  |  |  |  |  |  |  |
| **All patients** |  |  |  |  |  |  |  |  |  |  |  |  |  |  |  |
| VF (n) | 265 | 337 | 376 | 389 | 371 | 343 | 321 | 202 | 237 | 224 | 212 | 201 | 216 | 266 |  |
| Non missing (n) | 580 | 677 | 765 | 816 | 780 | 759 | 733 | 492 | 634 | 596 | 534 | 553 | 572 | 632 |  |
| VF ((%) | 46 | 50 | 49 | 48 | 48 | 45 | 44 | 41 | 37 | 38 | 40 | 36 | 38 | 42 | < 0.0001 |
| Missing (n) | 157 | 110 | 85 | 80 | 79 | 65 | 81 | 38 | 55 | 39 | 56 | 55 | 42 | 69 |  |
| **Bystander witnessed** |  |  |  |  |  |  |  |  |  |  |  |  |  |  |  |
| VF (n) | 196 | 251 | 290 | 282 | 283 | 263 | 236 | 168 | 183 | 167 | 163 | 162 | 184 | 212 |  |
| Non missing (n) | 347 | 421 | 514 | 515 | 480 | 462 | 454 | 327 | 404 | 345 | 321 | 338 | 378 | 423 |  |
| VF (%) | 56 | 60 | 56 | 55 | 59 | 57 | 52 | 51 | 45 | 48 | 51 | 48 | 49 | 50 | < 0.0001 |
| Missing (%) | 85 | 53 | 53 | 44 | 39 | 40 | 53 | 26 | 33 | 17 | 32 | 36 | 27 | 42 |  |
| **Non witnessed** |  |  |  |  |  |  |  |  |  |  |  |  |  |  |  |
| VF (n) | 41 | 54 | 60 | 80 | 61 | 65 | 61 | 25 | 37 | 42 | 32 | 32 | 21 | 42 |  |
| Non missing (n) | 161 | 183 | 191 | 233 | 236 | 242 | 222 | 136 | 168 | 202 | 172 | 175 | 155 | 168 |  |
| VF (%) | 25 | 30 | 31 | 34 | 26 | 27 | 27 | 18 | 22 | 21 | 19 | 18 | 14 | 25 | < 0.0001 |
| Missing (%) | 45 | 39 | 17 | 23 | 25 | 16 | 19 | 9 | 10 | 13 | 13 | 11 | 14 | 16 |  |
| **Delay** |  |  |  |  |  |  |  |  |  |  |  |  |  |  |  |
| From cardiac arrest to defibrillation among patients found in ventricular fibrillation (median, min) | 12 | 10 | 11 | 12 | 11 | 11 | 11 | 11 | 10 | 10 | 10 | 11 | 11 | 10 | 0.03 |
| Non missing (n) | 159 | 214 | 245 | 230 | 240 | 226 | 200 | 134 | 146 | 144 | 131 | 138 | 151 | 176 |  |
| Missing (n)** | 106 | 123 | 131 | 159 | 131 | 117 | 121 | 68 | 91 | 80 | 81 | 63 | 65 | 90 |  |
| **Survival** |  |  |  |  |  |  |  |  |  |  |  |  |  |  |  |
| **Patients found in VF** |  |  |  |  |  |  |  |  |  |  |  |  |  |  |  |
| Survival to 1 month (n) | 40 | 42 | 58 | 54 | 45 | 41 | 41 | 32 | 28 | 24 | 35 | 44 | 44 | 71 |  |
| Non missing (n) | 261 | 336 | 371 | 387 | 370 | 343 | 321 | 202 | 236 | 220 | 203 | 194 | 212 | 263 |  |
| Survival to 1 month (%) | 15.3 | 12.5 | 15.6 | 14.0 | 12.2 | 12.0 | 12.8 | 15.8 | 11.9 | 10.9 | 17.2 | 22.7 | 20.8 | 27.0 | < 0.0001 |
| Missing (n) | 4 | 1 | 5 | 2 | 1 | 0 | 0 | 0 | 1 | 4 | 9 | 7 | 4 | 3 |  |
| **Patients not found in VF** |  |  |  |  |  |  |  |  |  |  |  |  |  |  |  |
| Survival to 1 month (n) | 7 | 15 | 12 | 18 | 11 | 12 | 10 | 8 | 5 | 4 | 11 | 11 | 12 | 18 |  |
| Non missing (n) | 312 | 333 | 387 | 425 | 405 | 416 | 410 | 289 | 396 | 367 | 316 | 346 | 347 | 362 |  |
| Survival to 1 month (%) | 2.2 | 4.5 | 3.1 | 4.2 | 2.7 | 2.9 | 2.4 | 2.8 | 1.3 | 1.1 | 3.5 | 3.2 | 3.5 | 5.0 | > 0.2 |
| Missing (n) | 3 | 7 | 2 | 2 | 4 | 0 | 2 | 1 | 1 | 5 | 6 | 6 | 9 | 4 |  |

* p-value denoted if < 0.2

** Number of patients with missing information including all non witnessed cases

**Table 3**

**Total witnessed status, bystander CPR and outcome in relation to witnessed status**

|  | **1992** | **1993** | **1994** | **1995** | **1996** | **1997** | **1998** | **1999** | **2000** | **2001** | **2002** | **2003** | **2004** | **2005** | **p-value*** |
| --- | --- | --- | --- | --- | --- | --- | --- | --- | --- | --- | --- | --- | --- | --- | --- |
| **Witnessed status** |  |  |  |  |  |  |  |  |  |  |  |  |  |  |  |
| Bystander witnessed (n) | 432 | 474 | 567 | 559 | 519 | 502 | 507 | 353 | 437 | 362 | 353 | 374 | 405 | 465 |  |
| Non missing (n) | 638 | 696 | 775 | 815 | 780 | 760 | 748 | 498 | 615 | 577 | 538 | 560 | 574 | 649 |  |
| Bystander witnessed ((%) | 68 | 68 | 73 | 69 | 67 | 66 | 68 | 71 | 71 | 63 | 66 | 67 | 71 | 72 | >0.2 |
| Missing (n) | 99 | 91 | 75 | 81 | 79 | 64 | 66 | 32 | 74 | 58 | 52 | 48 | 40 | 52 |  |
| **Bystander CPR** |  |  |  |  |  |  |  |  |  |  |  |  |  |  |  |
| Bystander CPR (n) | 329 | 359 | 412 | 432 | 419 | 400 | 396 | 253 | 321 | 320 | 275 | 320 | 346 | 398 |  |
| Non missing (n) | 704 | 752 | 809 | 862 | 828 | 793 | 796 | 513 | 652 | 608 | 575 | 584 | 598 | 687 |  |
| Bystander CPR (%) | 47 | 48 | 51 | 50 | 51 | 50 | 50 | 49 | 49 | 53 | 48 | 55 | 58 | 58 | < 0.0001 |
| Missing (%) | 33 | 35 | 41 | 34 | 31 | 31 | 18 | 17 | 37 | 27 | 15 | 84 | 16 | 14 |  |
| **Survival** |  |  |  |  |  |  |  |  |  |  |  |  |  |  |  |
| **Bystander witnessed (n)** |  |  |  |  |  |  |  |  |  |  |  |  |  |  |  |
| Survival to 1 month (n) | 47 | 49 | 66 | 61 | 43 | 45 | 46 | 36 | 31 | 26 | 48 | 56 | 52 | 86 |  |
| Non missing (n) | 428 | 467 | 560 | 557 | 515 | 502 | 506 | 353 | 435 | 356 | 342 | 361 | 395 | 458 |  |
| VF (%) | 11.0 | 10.5 | 11.8 | 11.0 | 8.4 | 9.0 | 9.1 | 10.2 | 7.1 | 7.3 | 14.0 | 15.5 | 13.2 | 18.8 | 0.0007 |
| Missing (%) | 4 | 7 | 7 | 2 | 4 | 0 | 1 | 0 | 2 | 6 | 11 | 13 | 10 | 7 |  |
| **Non witnessed (n)** |  |  |  |  |  |  |  |  |  |  |  |  |  |  |  |
| Survival to 1 month (n) | 5 | 8 | 8 | 11 | 15 | 7 | 10 | 3 | 1 | 3 | 2 | 5 | 5 | 8 |  |
| Non missing (n) | 204 | 217 | 207 | 253 | 260 | 258 | 240 | 145 | 178 | 213 | 181 | 183 | 164 | 181 |  |
| Survival to 1 month (%) | 2.4 | 3.7 | 3.9 | 4.4 | 5.8 | 2.7 | 4.2 | 2.1 | 0.6 | 1.4 | 1.1 | 2.7 | 3.0 | 4.4 | > 0.2 |
| Missing (n) | 2 | 5 | 1 | 3 | 1 | 0 | 1 | 0 | 0 | 2 | 4 | 3 | 5 | 3 |  |
